# Evaluation of novel inflammatory biomarkers in overweight, obese, and morbidly obese children: a cross-sectional study

**DOI:** 10.3389/fendo.2026.1778022

**Published:** 2026-03-11

**Authors:** Semra Bahar Akin, Taha Yasin Akin

**Affiliations:** 1 Health Sciences University Derince Training and Research Hospital, Department of Pediatrics, Division of Pediatric Endocrinology, Derince, Kocaeli, Türkiye; 2 Division of Pediatric Allergy and Immunology, Faculty of Medicine, Kocaeli University, Kocaeli, Türkiye

**Keywords:** children, complete blood count, inflammatory indices, insulin resistance, monocyte-to-HDL-C ratio, obesity, systemic immune-inflammation index

## Abstract

**Background:**

Chronic low-grade inflammation (metaflammation) is a central feature of obesity and contributes to metabolic complications. Complete blood count (CBC)–derived inflammatory indices have been proposed as accessible markers of obesity-related inflammation, but their biological meaning in pediatric obesity remains unclear.

**Objective:**

To determine whether CBC-derived composite inflammatory indices primarily reflect adiposity-related inflammation or metabolic impairment in children with different obesity severities.

**Methods:**

This retrospective cross-sectional study included 417 children aged 5–18 years, categorized into four groups according to body mass index (BMI) percentiles: normal weight, overweight, obese, and morbidly obese. The CBC-derived inflammatory indices, including the systemic immune-inflammation index (SII), systemic inflammation response index (SIRI), aggregate inflammation index (AISI), and monocyte-to-high-density lipoprotein cholesterol ratio (MHR), were calculated. Insulin resistance was assessed using the Homeostasis Model Assessment for Insulin Resistance (HOMA-IR). Trend, correlation analyses were performed. Multivariate linear regression models based on a predefined clinical rationale were created, and their discriminative performances were evaluated using ROC analyses.

**Results:**

SII, SIRI, AISI, and MHR levels increased as the severity of obesity increased, and a significant difference was observed, especially between the normal weight and morbidly obese groups. In multivariate models, the significant relationship between BMI-SDS and SII, AISI, and MHR persisted, whereas no relationship was found with SIRI. HOMA-IR was only associated with MHR. In ROC analyses, the ability of the inflammatory indices to distinguish between morbid obesity and insulin resistance was found to be limited (AUC approximately 0.55–0.68).

**Conclusion:**

CBC-derived inflammatory indices in pediatric obesity appear to primarily reflect adiposity-related inflammatory burden rather than isolated insulin resistance. However, owing to their limited explanatory power and low discriminative performance, they should not be interpreted as standalone diagnostic tools but as biomarkers supporting clinical assessment.

## Introduction

Obesity is a persistent and complex disease marked by an excessive buildup of adipose tissue, which negatively influences health in numerous ways. In recent years, the marked increase in the prevalence of overweight and obesity during childhood and adolescence has made this condition a critical public health issue of global significance. Obesity that begins at an early age is a strong determinant of lifelong cardiometabolic risk and constitutes the primary starting point for metabolic disorders in adulthood. In a large-scale analysis by Gao et al. covering 191 countries, the prevalence of overweight and obesity in the 5–19 age group rose from 4% in 1975 to over 18% in 2016, and the current trend is expected to continue until 2030 (1). This significant increase has led to a parallel increase in obesity-related metabolic complications. Insulin resistance (IR), dyslipidemia, non-alcoholic fatty liver disease, and type 2 diabetes are among the most common metabolic outcomes observed in pediatric obesity (2–4).

The fundamental pathophysiological link between obesity and its metabolic complications is chronic low-grade inflammation, also referred to as metaflammation. The inflammation that originates in adipose tissue leads to an increase in systemic inflammation due to the release of proinflammatory cytokines like TNF-α, IL-6, and leptin from the tissue, which in turn disrupts insulin signaling (5–9). This subclinical inflammatory process may also be reflected through changes in peripheral blood cell counts, and hematological parameters can be considered potential indicators of metabolic risk (10, 11).

A complete blood count (CBC) is an easily accessible and low-cost test that allows for the quantitative evaluation of cell series, such as leukocytes, lymphocytes, monocytes, and platelets, which play a role in inflammatory processes. Classical inflammatory ratios derived from CBC, such as the neutrophil to lymphocyte ratio (NLR) and platelet to lymphocyte ratio (PLR), have been associated with metabolic syndrome and insulin resistance (12, 13). In recent years, several composite indices have been developed to more comprehensively reflect the systemic inflammatory response. These include the systemic immune-inflammation index (SII = [platelets × neutrophils]/lymphocytes), the systemic inflammation response index (SIRI = [neutrophils × monocytes]/lymphocytes), the aggregate index of systemic inflammation (AISI = [neutrophils × monocytes × platelets]/lymphocytes), and the monocyte/HDL-C ratio (MHR) (14–25). Although studies have shown that these indices are associated with metabolic syndrome and cardiovascular diseases in the adult population, available data on pediatric obesity are limited.

In this study, the relationships between hemogram-derived composite inflammatory indices (SII, SIRI, AISI, and MHR) and both adiposity and insulin resistance were examined in children with different obesity severities. The main hypothesis of the study is that, in pediatric obesity, the primary determinant of the inflammatory response is adiposity rather than metabolic impairment; therefore, the associations of the inflammatory indices with BMI-SDS will persist even after adjustment for insulin resistance. Therefore, the relationships between inflammatory indices and BMI-SDS were evaluated after adjusting for insulin resistance.

## Methods

### Study design and participants

**Figure 1:**
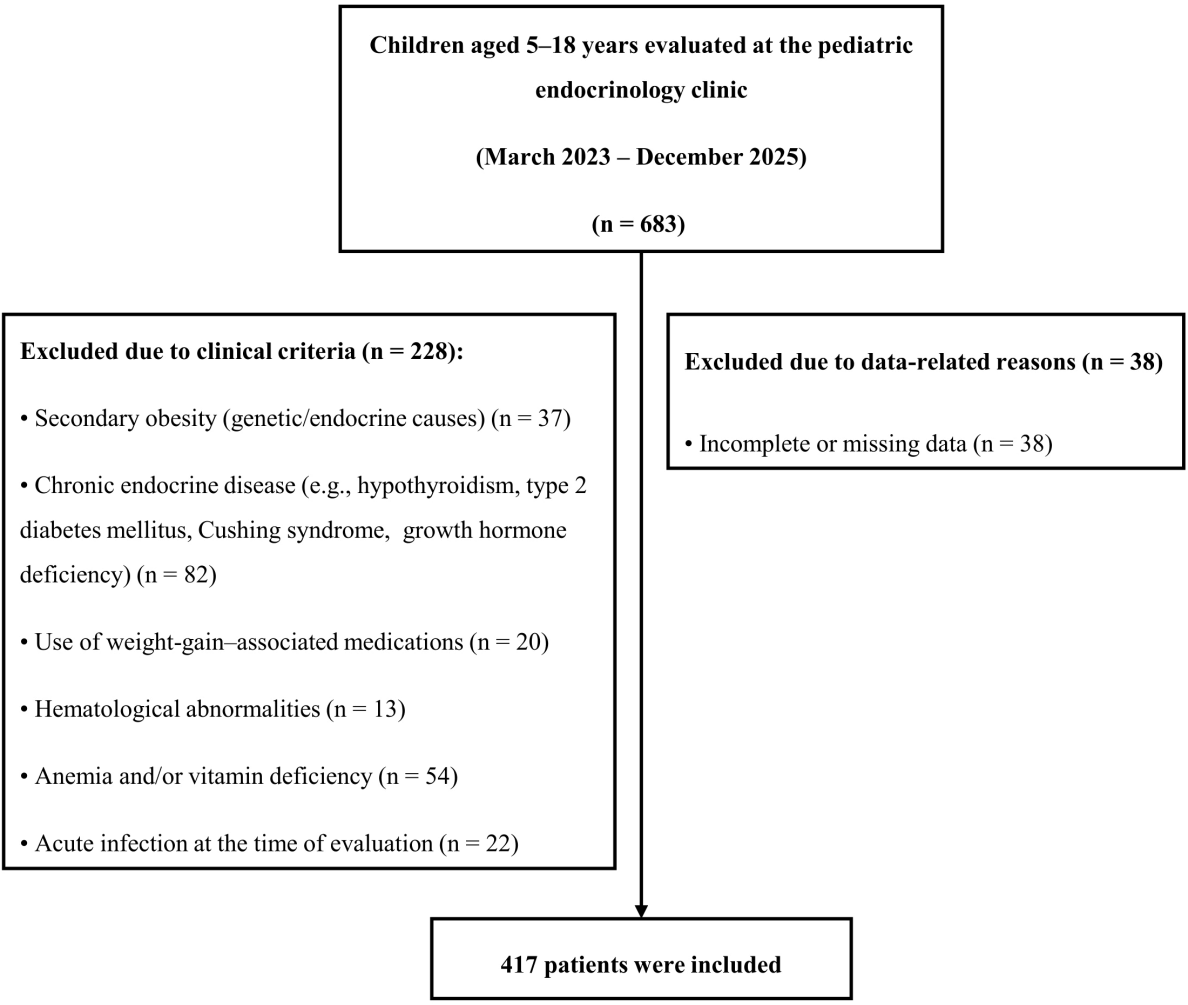
Flow diagram of participant selection.

This retrospective cross-sectional study encompassed children and adolescents aged 5 to 18 years who were assessed at the Pediatric Endocrinology Clinic of our tertiary care center between March 2023 and December 2025. Children younger than 5 years were excluded because early childhood is characterized by distinct physiological insulin sensitivity and hematological profiles, which may limit the interpretability of CBC-derived inflammatory indices. The 5–18 year age range was therefore selected to encompass both childhood and adolescence, allowing evaluation of obesity-related inflammatory changes across prepubertal and pubertal stages, which represent the population routinely followed in pediatric endocrinology practice. Patients with secondary obesity (genetic, monogenic, or endocrine causes), hypertension, chronic disease, acute infection, liver, kidney, or thyroid dysfunction, hematological abnormalities (leukopenia <4.0 ×10³/µL, leukocytosis >13.0 ×10³/µL, anemia or thrombocytopenia <150 ×10³/µL), use of medications that may affect complete blood count parameters or body weight, and those with incomplete data were excluded from the study. Patients with anemia and vitamin deficiency were also excluded. Participant selection was conducted in accordance with STROBE recommendations and is summarized in **Figure 1**. The normal-weight control group was recruited from a tertiary pediatric endocrinology clinic rather than from the general population. In our healthcare referral system, a substantial proportion of children evaluated in pediatric endocrinology clinics are referred because of transient, borderline, or suspected laboratory abnormalities identified during routine screening and are ultimately found to have normal clinical and laboratory evaluations.

The patients included in the study were divided into four groups according to their BMI percentiles. Those with a BMI in the 85th–95th percentile range were classified as overweight, those with a BMI ≥95th percentile as obese, and those with a BMI ≥99th percentile as morbidly obese. Children with a BMI below the 85th percentile was included in the normal weight control group (26). These national BMI reference curves are broadly comparable with World Health Organization (WHO) and Centers for Disease Control and Prevention (CDC) growth standards in terms of percentile-based classification of overweight and obesity, while providing population-specific accuracy for Turkish children (27, 28).

The normal-weight control group was recruited from children evaluated at the same tertiary pediatric endocrinology clinic as the obesity groups. In our national healthcare system, pediatric endocrinology clinics frequently receive referrals for children with transient or borderline laboratory findings detected during routine primary care screening, as well as referrals made without a confirmed endocrine pathology.

All participants included in the control group had normal anthropometric measurements and normal metabolic and hematological laboratory findings at the time of evaluation and had no diagnosed endocrine or chronic disease.

All data were obtained retrospectively from clinical records, and no additional blood samples were collected for research. The institution’s ethics committee granted approval for the study protocol, which was carried out in line with the principles outlined in the Declaration of Helsinki.

### Pubertal status and anthropometric measurements

Pubertal status was evaluated utilizing the Tanner staging system. Males with a testicular volume of ≥4 mL and females exhibiting breast development at Tanner stage 2 or higher were classified as pubertal, whereas other individuals were considered prepubertal (29). Height was measured using a Harpenden fixed stadiometer, and body weight was determined using a mechanical scale (Seca GmbH & Co. KG, Hamburg, Germany). The BMI was calculated by dividing body weight in kilograms by the square of height in meters (kg/m²).

### Clinical and laboratory data

Demographic and clinical data were obtained from patient files, and age, sex, and pubertal status were recorded. All blood samples, including hemogram, serum glucose, lipid parameters (triglycerides, high-density lipoprotein cholesterol [HDL-C], low-density lipoprotein cholesterol [LDL-C], and total cholesterol [TC]), aspartate aminotransferase (AST), alanine aminotransferase (ALT), ferritin, folate, vitamin B12, HbA1c, and insulin levels, were collected after a 12-hour overnight fasting period as part of routine clinical evaluation.

Serum glucose, lipid parameters (triglycerides, high-density lipoprotein cholesterol (HDL-C), low-density lipoprotein cholesterol (LDL-C), and total cholesterol), aspartate aminotransferase (AST), and alanine aminotransferase (ALT) levels were measured as part of routine clinical practice at our hospital using a fully automated spectrophotometric analyzer (Mindray BS-2000; Shenzhen, China). Serum glucose was determined using the glucose oxidase–peroxidase (GOD–POD) method, triglycerides using the glycerokinase–peroxidase method, total cholesterol using the cholesterol oxidase–peroxidase (CHOD–POD) method, and HDL-C and LDL-C using direct enzymatic colorimetric assays. AST and ALT activities were assessed using ultraviolet kinetic methods, without pyridoxal phosphate activation. Serum insulin was measured using a chemiluminescent microparticle immunoassay (CMIA) with the ARCHITECT i8000 system (Abbott Diagnostics, Abbott Park, IL, USA). Glycated hemoglobin (HbA1c) levels were determined using high-performance liquid chromatography (HPLC) with a Lifotronic H100 analyzer.

All laboratory analyses were performed in the same hospital laboratory using standardized, routinely calibrated automated analyzers in accordance with internal quality control procedures.

### Definition of insulin resistance

Insulin resistance was defined using sex- and pubertal stage–specific homeostasis model assessment of insulin resistance (HOMA-IR) cut-off values validated in a Turkish pediatric population. Based on the reference study by Kurtoğlu et al., insulin resistance was defined as HOMA-IR ≥ 2.67 in prepubertal boys and ≥ 5.22 in pubertal boys, and ≥ 2.22 in prepubertal girls and ≥ 3.82 in pubertal girls (30). Pubertal status was determined using Tanner staging and applied across the entire age range of 5–18 years. HOMA-IR was calculated by dividing the product of fasting glucose (mg/dL) and fasting insulin (µU/mL) levels by 405.

### Calculation of inflammation indexes

The CBC parameters, including WBC, PLT, PDW, MPV, and PCT, along with WBC differentials such as neutrophils, lymphocytes, and monocytes, were assessed using flow impedance, laser light scattering, and flow cytometry (Mindray, BC2000). To assess systemic inflammation, several indices were calculated from the CBC parameters: the systemic immune-inflammation index (SII = [platelet × neutrophil]/lymphocyte), the systemic inflammation response index (SIRI = [neutrophil × monocyte]/lymphocyte), the aggregate systemic inflammation index (AISI = [neutrophil × monocyte × platelet]/lymphocyte), and the monocyte/high-density lipoprotein cholesterol ratio (MHR = monocyte/HDL-C). Additionally, traditional indices like the neutrophil-to-lymphocyte ratio (NLR) and the platelet-to-lymphocyte ratio (PLR) were also analyzed.

### Statistical analysis

All statistical analyses were conducted utilizing IBM SPSS for Windows version 29.0 (IBM Corp., Armonk, NY, USA). The Kolmogorov–Smirnov and Shapiro–Wilk tests were employed to evaluate the assumption of normality. Continuous variables are presented as median and interquartile range (IQR) due to the non-fulfillment of the normality assumption, while categorical variables are reported as frequencies and percentages. Group comparisons were performed using the Mann–Whitney U test. Associations between categorical variables were analyzed using the chi-square test with Bonferroni correction. Correlations between continuous variables were assessed using Spearman’s rank correlation coefficient. Monotonic trends across ordinal categories were evaluated using the Jonckheere–Terpstra test.

Pre-specified multivariable linear regression models were constructed based on clinical rationale to examine the associations between inflammatory indices and metabolic parameters. All variables of interest were entered simultaneously using the enter method. Log-transformation was applied before modeling. Multicollinearity was evaluated using variance inflation factors, and model assumptions were assessed using residual diagnostics.

No imputation was performed for missing data. Multivariable analyses were conducted using complete-case analysis including only participants with available values for all variables in the model.

Study outcomes were prespecified as primary, secondary, or exploratory. The primary outcome was the association between BMI-SDS and the predefined systemic inflammatory indices (SII, SIRI, AISI, and MHR), which were analyzed as related markers reflecting a common inflammatory construct. Comparisons according to insulin resistance status were considered secondary analyses. Correlation and multivariable regression analyses were exploratory.

To account for multiple comparisons across the predefined primary outcomes, the false discovery rate was controlled using the Benjamini–Hochberg procedure, and adjusted p-values are reported as q values. No multiple testing correction was applied to secondary or exploratory analyses.

Receiver operating characteristic (ROC) curve analysis was performed for the purpose of evaluating diagnostic performance. The power of inflammatory indices to distinguish morbid obesity and insulin resistance was assessed using the area under the curve (AUC). The most appropriate cutoff values were determined using the Youden index.

A p-value < 0.05 was considered statistically significant.

## Results

During the study period, 683 children aged 5–18 years who were evaluated at the pediatric endocrinology outpatient clinic were screened for eligibility. Due to pre-determined clinical exclusion criteria, 228 patients were excluded from the study: secondary obesity (n=37), chronic endocrine diseases (n=82), medication use associated with weight gain (n=20), hematological abnormalities (n=13), anemia and/or vitamin deficiency (n=54), and acute infection (n=22). In addition, 38 patients were excluded due to missing or insufficient data. Consequently, 417 children were included in the analyses (**Figure 1**). Of these, 121 were of normal weight (40 boys; median age, 11.2 years), 91 were overweight (32 boys; median age, 11.7 years), 113 were obese (43 boys; median age, 11.7 years), and 92 were morbidly obese (28 boys; median age, 12.6 years). Insulin resistance was absent in 226 (54.2%) patients, while it was present in 191 (45.8%) (**Supplementary Table 1**). Prediabetes was identified in 76 (18.2%) participants in the study population.

There were no significant differences between the groups in terms of age, sex distribution, or pubertal status (**Table 1**). HOMA-IR and fasting insulin levels were highest in the morbidly obese group and lowest in the normal weight group (p<0.001; **Table 1**). HDL cholesterol levels were lowest in the morbidly obese group and highest in the normal weight group, with a significant difference observed between the groups (p = 0.002; **Table 1**).

**Table 1:** Demographic, anthropometric, and metabolic characteristics of the study population according to obesity categories.

Variable	Normal (n=121)	Overweight (n=91)	Obese (n=113)	Morbid Obese (n=92)	*p*-value
**Age (years)**	11.2 (9.2–14.7)	11.7 (9.2–14.5)	11.7 (9.0–14.0)	13.6 (9.7–15.3)	0.204
**Female Gender (%)**	81 (66.9)	59 (64.8)	70 (61.9)	63 (69.2)	0.724
**Pubertal (%)**	79 (65.3)	64 (70.3)	73 (64.6)	62 (68.1)	0.812
**Weight (kg)**	37.5 (26–48)	59 (42–73)	67.5 (49–82.75)	85 (67–103)	**<0.001**
**Weight SDS**	–0.49 (–1.25–0.41)	1.90 (1.33–2.29)	2.56 (2.18–3.02)	3.59 (3.13–4.41)	**<0.001**
**Height (cm)**	143 (130.5–155)	151 (140–166)	153 (137–165)	159 (145–165)	**<0.001**
**Height SDS**	–0.48 (–1.47–0.49)	0.64 (–0.08–1.61)	0.80 (0.01–1.65)	0.72 (–0.12–1.48)	**<0.001**
**BMI**	17.6 (15.9–20.1)	24.9 (22.5–27.0)	28.3 (25.5–30.5)	33.0 (31.1–37.2)	**<0.001**
**BMI SDS**	–0.24 (–1.07–0.44)	1.85 (1.49–2.02)	2.42 (2.25–2.60)	3.06 (2.93–3.60)	**<0.001**
**HOMA-IR**	2.0 (1.1–2.4)	3.1 (2.2–4.6)	4.2 (2.7–5.5)	4.6 (3.2–7.6)	**<0.001**
**Insulin (µIU/mL)**	8.3 (5–12)	14.3 (10.2–18.7)	17.5 (11.3–23.9)	19.6 (14.5–32.8)	**<0.001**
**Glucose (mg/dL)**	89.5 (82–97)	93 (89–98.3)	94 (89–100.3)	93 (88–101.5)	**0.001**
**HbA1c (%)**	5.2 (5–5.5)	5.3 (5.1–5.5)	5.4 (5.2–5.6)	5.4 (5.2–5.6)	**0.01**
**Total Cholesterol (mg/dL)**	147.5 (133.8–170.8)	166.5 (144.0–189.3)	159.0 (138.0–174.5)	159.0 (141.0–175.5)	0.109
**LDL Cholesterol (mg/dL)**	79 (68.75–99.75)	93.3 (77–112.75)	88 (70–104)	89 (73–107)	0.146
**HDL Cholesterol (mg/dL)**	56 (51–61)	49 (44–57)	48 (43–55)	47 (41–57)	**0.002**
**Triglycerides (mg/dL)**	65 (55–96)	114 (78–142)	101 (71–136)	96 (71–131)	**0.001**
**ALT (U/L)**	14 (12–18)	19 (15–28)	23 (18–30.5)	23 (16.45–30.5)	**<0.001**
**AST (U/L)**	24 (19–29)	22 (18.5–26)	23 (19–30)	23 (18.75–28)	0.474

BMI, body mass index; SDS, standard deviation score; HOMA-IR, Homeostatic Model Assessment for Insulin Resistance; TC, total cholesterol; TG, triglycerides; HDL-C, high-density lipoprotein cholesterol; LDL-C, low-density lipoprotein cholesterol; HbA1c, glycated hemoglobin; AST, aspartate aminotransferase; ALT, alanine aminotransferase.

Bold values indicate statistical significance (p < 0.05).

When the hematological parameters were examined, differences were observed in the levels of WBC, NEU, RBC, MCV, RDW, MCH, and MCHC (all p < 0.001; **Table 2**).

**Table 2:** Hematological parameters of the study population according to obesity categories.

Variable	Normal	Overweight	Obese	Morbid Obese	*p*-value
**WBC (10³/µL)**	7.3 (6.3–8.2)	8.2 (7–9.6)	8.1 (6.7–9.4)	8.6 (7.1–10.0)	**<0.001**
**NE (10³/µL)**	3.6 (2.9–4.2)	4.2 (3.5–5.3)	3.9 (3.2–5.2)	4.7 (3.7–5.6)	**<0.001**
**LY (10³/µL)**	2.8 (2.3-3.3)	2.9 (2.6-3.6)	2.9 (2.4-3.4)	3 (2.5-3.5)	0.497
**MO (10³/µL)**	0.6 (0.5-0.7)	0.6 (0.5-0.8)	0.6 (0.5-0.7)	0.6 (0.5-0.8)	0.322
**PLT (10³/µL)**	309 (270-362)	330 (291-377)	336 (278-385)	332 (289-386)	0.052
**HGB (g/dl)**	12.7 (12.1-13.4)	13 (12.5-13.7)	12.8 (12.3-13.5)	12.9 (12.0-13.6)	0.094
**RBC (10^6^/µL)**	4.7 (4.5–4.9)	4.9 (4.6–5.2)	4.9 (4.7–5.2)	4.9 (4.6–5.1)	**<0.001**
**MCV (fL)**	83.3 (81.2–86.0)	82.2 (80.0–85.7)	80.3 (77.8–83.4)	80.8 (77.3–84.1)	**<0.001**
**RDW (%)**	12.7 (12.3–13.5)	13.2 (12.5–13.8)	13.5 (12.9–14.8)	14 (13.2–14.8)	**<0.001**
**MCH**	27.7 (26.3–28.9)	27.2 (26.1–28.7)	26.2 (25.0–27.8)	26.3 (25.2–27.7)	**<0.001**
**MCHC**	33.0 (32.5–33.8)	33.0 (32.4–33.6)	32.7 (31.9–33.7)	32.5 (31.7–33.3)	**<0.001**

Bold values indicate statistical significance (p < 0.05).

**Figure 2:**
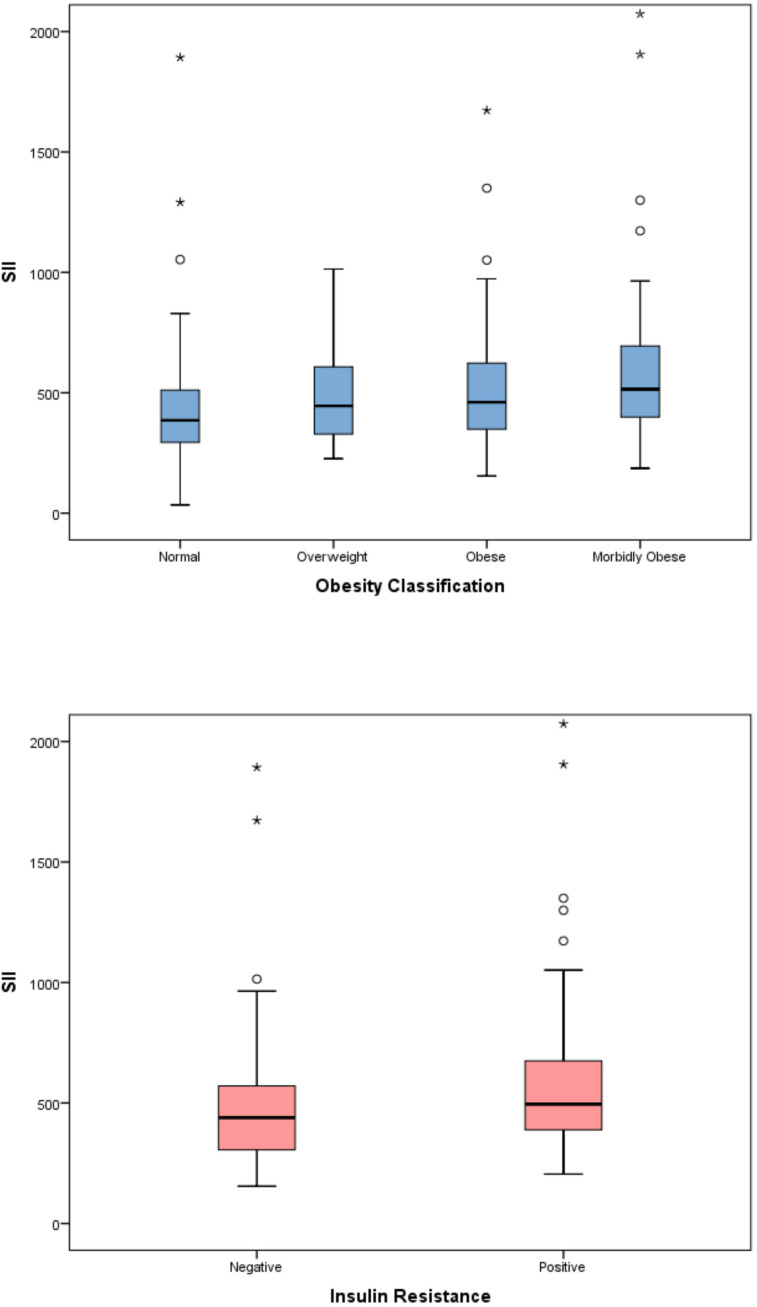
Distribution of the systemic immune-inflammation index (SII) across obesity categories in children.

**Figure 3:**
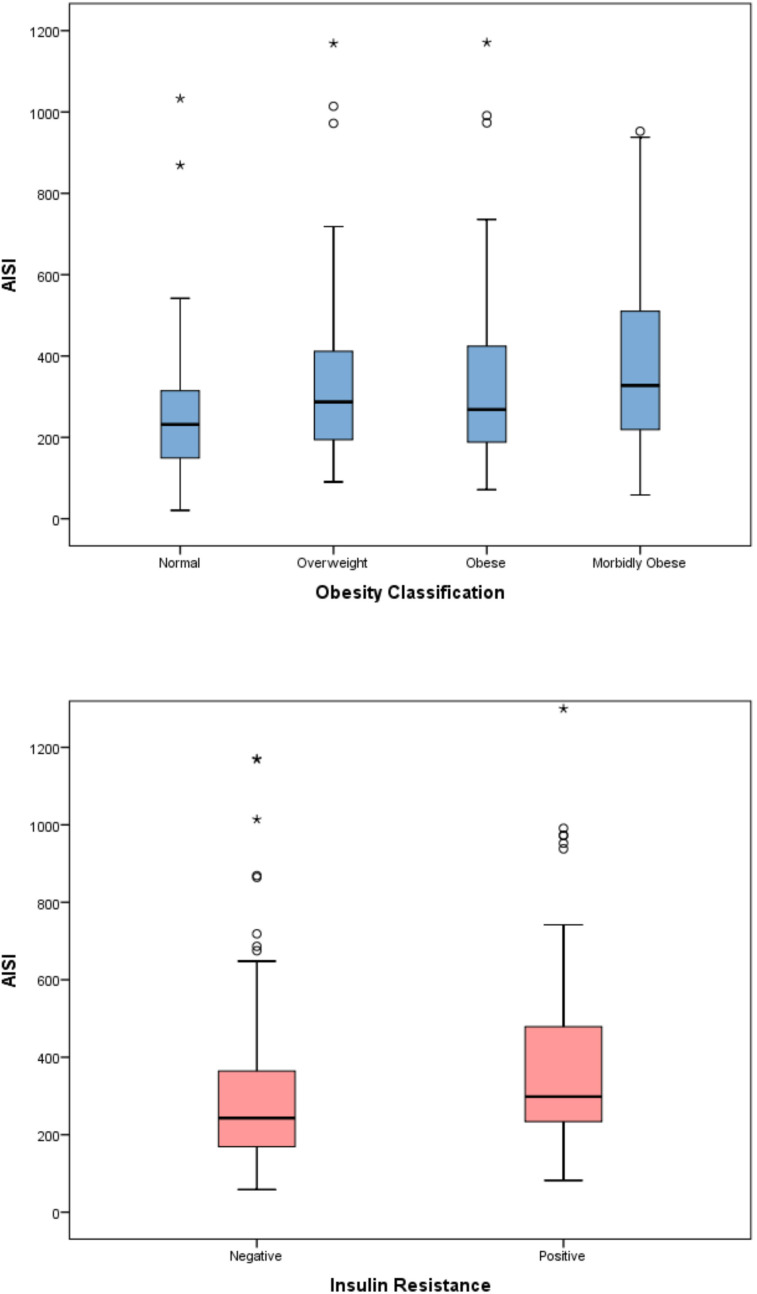
Distribution of the aggregate index of systemic inflammation (AISI) according to obesity severity.

**Figure 4:**
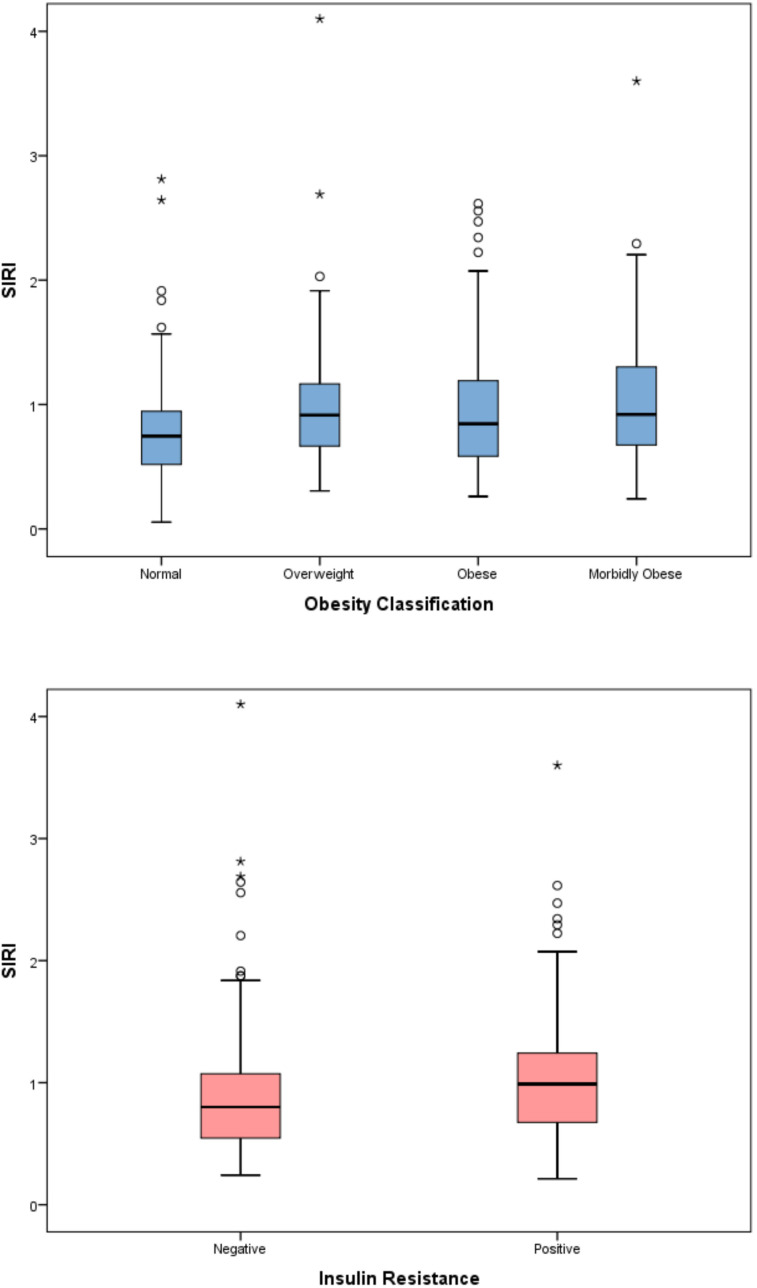
Distribution of the systemic inflammation response index (SIRI) across BMI-defined obesity groups.

**Figure 5:**
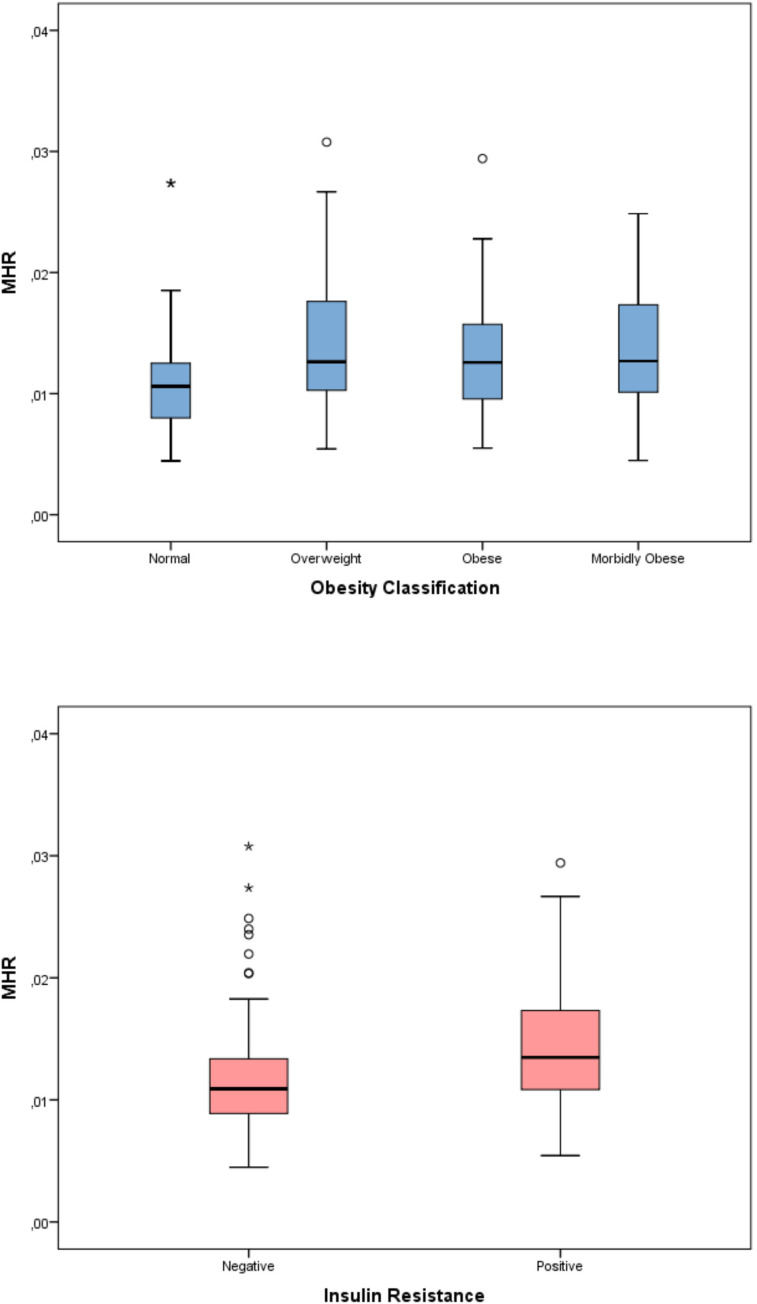
Monocyte-to-HDL cholesterol ratio (MHR) according to obesity status and insulin resistance.

The evaluation of inflammatory indices showed significant differences among the four obesity categories in terms of SII, SIRI, and AISI (p < 0.001 for all parameters). In the *post-hoc* analysis with pairwise comparisons, a statistically significant difference persisted only between the normal and morbidly obese groups. Although a significant difference was detected among the four obesity categories for the MHR parameter (p=0.01), this significance disappeared in the pairwise comparisons of the *post-hoc* analysis. The significance of SII, SIRI, and AISI persisted even after applying the Benjamini–Hochberg multiple comparison correction for the predefined primary endpoints (all q < 0.05) (**Table 3**) (**Figures 2**–**5**). Trend analysis revealed a statistically significant upward trend in SII, SIRI, AISI, and MHR, whereas no significant trend was observed for NLR, and PLR. These trends did not change after multiple comparison corrections (**Table 4**).

**Table 3:** Significant inflammatory indices across obesity groups.

Variable	Normal	Overweight	Obese	Morbid Obese	*p*- value	q-value
SII*	385.5 (293.5-512.1)	445.2 (324.0-609.8)	460.9 (346.6-632.5)	515.0 (398.4-705.2)	**<0.001**	**0.000212**
AISI*	231.7 (148.8–314.8)	287.3 (192.8–413.2)	268.4 (187.0–424.7)	327.5 (218.9–522.7)	**<0.001**	**0.000256**
SIRI*	0.75 (0.52–0.95)	0.92 (0.67–1.17)	0.85 (0.58–1.20)	0.92 (0.68–1.31)	**<0.001**	**0.001060**
MHR**	10.6 (7.6-12.5)	12.6 (10.2-17.7)	12.6 (9.6-15.8)	12.7 (10.1-17.4)	**0.01**	**0.009709**
**NLR**	1.26 (0.95-1.63)	1.41 (1.08-1.93)	1.37 (1.07-2.00)	1.60 (1.16-1.85)	0.900	*NA*
**PLR**	112.7 (91.1–132.5)	109.7 (87.2-133.3)	115.4 (93.9-137.9)	114.1 (92.4-144.1)	0.781	*NA*

* In the post-hoc analysis with Bonferroni correction, a statistically significant difference between the normal-weight and morbidly obese groups was observed for SII, SIRI, AISI. ** In the post-hoc analysis with Bonferroni correction, there is no significant difference between pairwise group.

q-values were calculated using the Benjamini–Hochberg procedure for predefined primary outcomes.

Bold values indicate statistical significance (p < 0.05).

**Table 4:** Trend analysis of inflammatory indices across BMI categories.

Inflammatory index	J–T Z statistic	p-value	q-value
SII	4.560	**<0.001**	**0.000020**
AISI	4.160	**<0.001**	**0.000064**
SIRI	3.723	**<0.001**	**0.000263**
MHR	2.027	**0.043**	**0.043**
WBC	4.356	**<0.001**	**NA**
NEU	5.163	**<0.001**	**NA**
PLT	2.626	**0.009**	**NA**
NLR	0.504	0.614	**NA**
PLR	0.988	**0.323**	**NA**

BMI categories were treated as an ordinal variable.

q-values were calculated using the Benjamini–Hochberg procedure for predefined primary outcomes.

Bold values indicate statistical significance (p < 0.05).

When the relationship between inflammatory indices and insulin resistance was evaluated, the SII, SIRI, AISI, and MHR indices were significantly higher in patients with insulin resistance (p<0.001 for all) (**Table 5**) (**Figures 2**–**5**).

**Table 5:** Inflammatory indices according to insulin resistance status.

Variable	Insulin resistance (–)	Insulin resistance (+)	p-value
**SII**	433.3 (304.6–563.9)	508.9 (388.9-694.6)	**<0.001**
**AISI**	237.6 (166.9–363.8)	319.2 (236.0–486.2)	**<0.001**
**SIRI**	0.793 (0.551–1.057)	1.000 (0.692–1.260)	**<0.001**
**MHR**	10.7 (8.8–13.3)	13.6 (11.1–17.4)	**<0.001**
**NLR**	105.9 (87.2–132.9)	115.9 (94.2–137.7)	0.124
**PLR**	114.5 (91.5–134.1)	113.8 (96.2–139.5)	0.502

Bold values indicate statistical significance (p < 0.05).

In the correlation analysis, statistically significant positive correlations were found between BMI SDS and SII, SIRI, AISI, and MHR (ρ = 0.221, 0.182, 0.206, and 0.213, respectively; all p < 0.001). HOMA-IR showed statistically significant positive correlations with SII, SIRI, AISI, and MHR (ρ = 0.252, 0.253, 0.294, and 0.383, respectively; all p < 0.001). (**Table 6**).

**Table 6:** Correlations between BMI, HOMA-IR, and inflammatory indices.

Inflammatory Index	BMI (SDS) ρ	*p* value	HOMA-IR ρ	*p* value
**NLR**	0.026	0.618	0.024	0.677
**PLR**	0.036	0.476	0.051	0.376
**SII**	0.221	<0.001	0.252	**<0.001**
**SIRI**	0.182	<0.001	0.253	**<0.001**
**AISI**	0.206	<0.001	0.294	**<0.001**
**MHR**	0.213	<0.001	0.383	**<0.001**

Bold values indicate statistical significance (p < 0.05).

In the multivariate linear regression models created for log-transformed inflammatory indices, evaluations were conducted together with age, sex, pubertal status, and HOMA-IR (**Table 7**). BMI-SDS was significantly and positively associated with SII (β=0.062, 95% CI 0.024–0.100, p=0.001). Similarly, a significant relationship was detected between BMI-SDS and both AISI (β=0.063, 95% CI 0.004–0.122, p=0.037) and MHR (β=0.053, 95% CI 0.005–0.101, p=0.032). In contrast, no significant relationship was observed between the BMI-SDS and SIRI. HOMA-IR was significantly and positively associated only with MHR (β=0.018, 95% CI 0.005–0.030, p=0.005) and did not show a significant relationship with the other inflammatory indices. No multicollinearity was detected in any of the models (VIF<2.5). In sensitivity analyses excluding HOMA-IR from the models, the direction and magnitude of the associations between BMI-SDS and inflammatory indices remained largely similar (**Supplementary Table 4**).

**Table 7:** Multivariable linear regression analyses of log-transformed inflammatory indices adjusted for pubertal status.

Variable	ln(SII) β (95% CI)	p value	ln(AISI) β (95% CI)	p value	ln(SIRI) β (95% CI)	p value	ln(MHR) β (95% CI)	p value
**BMI-SDS**	0.062 (0.024–0.100)	**0.001**	0.063 (0.004–0.122)	**0.037**	0.031 (–0.022–0.085)	**0.251**	0.053 (0.005–0.101)	**0.032**
**HOMA- IR**	0.001 (–0.012–0.014)	0.890	0.012 (–0.008–0.032)	0.242	0.010 (–0.008–0.028)	0.259	0.018 (0.005–0.030)	**0.005**
**Age**	0.004 (–0.018–0.026)	0.741	0.002 (–0.033–0.036)	0.925	0.016 (–0.015–0.048)	0.300	–0.004 (–0.028–0.019)	0.723
**Sex**	–0.131 (–0.238– –0.024)	**0.017**	–0.088 (–0.253–0.078)	0.299	–0.062 (–0.214–0.089)	0.421	0.034 (–0.077–0.146)	0.544
**Pubertal status**	0.127 (–0.034–0.288)	0.122	0.166 (–0.084–0.415)	0.193	0.120 (–0.108–0.348)	0.301	0.038 (–0.132–0.209)	0.660

All dependent variables were log-transformed prior to analysis due to skewed distributions. Multivariable linear regression models were pre-specified based on clinical rationale and constructed using the enter method. All models were adjusted for BMI-SDS, HOMA-IR, age, sex, and pubertal status. Variance inflation factors were <2.5 for all models, indicating no relevant multicollinearity. Adjusted R² values ranged from 0.03 to 0.07, indicating modest explanatory power.

Bold values indicate statistical significance (p < 0.05).

In subgroup analyses conducted to assess the possible effect-modifying role of pubertal status, no significant relationship was observed between inflammatory indices and BMI-SDS during the prepubertal period, whereas SII and AISI were significantly associated with HOMA-IR. In contrast, in the pubertal group, a significant relationship was detected between BMI-SDS and especially SII and MHR, whereas the association with HOMA-IR were not observed for other indices and persisted only for the MHR. (**Supplementary Tables 2**, **S3**).

The diagnostic performance for distinguishing morbid obesity is presented in **Table 8**. Among the inflammatory indices, the highest AUC value was found for SII (AUC = 0.623, 95% CI 0.573–0.672). This was followed by AISI (AUC = 0.607), SIRI (AUC = 0.597), and MHR (AUC = 0.553), respectively. At the cutoff points determined according to the Youden index, an SII threshold value of >428.3 demonstrated 70.6% sensitivity and 51.0% specificity, while an MHR threshold value of >0.017 showed 29.6% sensitivity and 82.8% specificity. The diagnostic performance of predicting insulin resistance is presented in **Table 9**. The highest AUC value was obtained for MHR (AUC = 0.679, 95% CI = 0.619–0.736). AISI (AUC = 0.655), SII (AUC = 0.626), and SIRI (AUC = 0.626) showed similar levels of performance. At the threshold value of MHR >0.013, the sensitivity was 61.6% and the specificity was 71.4%. Overall, the AUC values indicated only modest discriminative ability for both morbid obesity and insulin resistance, suggesting limited diagnostic utility when these indices are used alone (**Supplementary Figures 1–8**).

**Table 8:** Diagnostic performance of inflammatory indices for morbid obesity.

Index	AUC (95% CI)	Cut-off value	Sensitivity % (95% CI)	Specificity % (95% CI)
**SII**	0.623 (0.573–0.672)	>428.3	70.6 (59.7-80.0)	51.0 (45.2–56.8)
**AISI**	0.607 (0.556–0.656)	>318.24	51.8 (40.7-62.7)	65.9 (60.2-71.2)
**SIRI**	0.597 (0.547–0.647)	>1.0	47.1 (36.1-58.2)	71.2 (65.7-76.2)
**MHR**	0.553 (0.492–0.613)	>0.017	29.6 (20.0-40.8)	82.8 (76.7-87.9)

Cut-off value determined by the Youden index.

**Table 9:** Diagnostic performance of inflammatory indices for insulin resistance.

Index	AUC (95% CI)	Cut-off value	Sensitivity % (95% CI)	Specificity % (95% CI)
**SII**	0.626 (0.569–0.680)	>602.2	38.4 (30.8–46.4)	82.4 (75.3–88.2)
**AISI**	0.655 (0.599–0.708)	>232.8	77.4 (70.1–83.6)	48.7 (40.4–57.0)
**SIRI**	0.626 (0.570–0.681)	>0.95	55.4 (47.3–63.2)	66.9 (58.7–74.4)
**MHR**	0.679 (0.619–0.736)	>0.013	61.6 (53.2-69.6)	71.4 (62.1-79.6)

Cut-off value determined by the Youden index.

## Discussion

In our study, we demonstrated that with increasing obesity severity in children and adolescents, composite indices derived from CBC, such as SII, SIRI, and AISI, as well as the MHR index, which includes HDL in its formulation, were elevated. Pairwise comparisons revealed that this difference was mainly significant between normal-weight individuals and the morbidly obese group, whereas trend analyses showed a linear increase in these indices across patient groups. These findings support the idea that the inflammatory burden in pediatric obesity increases linearly with the severity of obesity. Additionally, our study showed the relationship between SII, SIRI, AISI, and MHR indices and insulin resistance, which is a metabolic complication of obesity. In multivariate analyses, it was observed that the relationship between BMI-SDS and inflammatory indices persisted for SII, AISI, and MHR, whereas the association with HOMA-IR remained only for MHR. Although threshold values could be determined in ROC analyses conducted to evaluate diagnostic discrimination, the AUC values were found to be limited. These findings suggest that, rather than serving as standalone clinical decision-making tools, the mentioned indices may be considered as complementary markers reflecting the inflammatory burden accompanying obesity.

Metaflammation, which arises alongside obesity, describes a chronic and low-grade systemic inflammatory response triggered by excessive nutrient intake, with adipose tissue being the main source of this process (8, 31). Although hypertrophy and hyperplasia in adipocytes in response to energy surplus are initially adaptive processes, exceeding the expansion capacity of adipocytes initiates pathological inflammation through mechanisms such as cellular hypoxia, mechanical stress, and endoplasmic reticulum stress. In this process, proinflammatory cytokines such as TNF-α, IL-6, and IL-1β released by adipocytes and stromal cells activate JNK and NF-κB signaling pathways, disrupting insulin receptor signaling and contributing to the development of systemic insulin resistance (10, 32, 33). Furthermore, in response to these proinflammatory cytokines secreted by adipocytes, monocytes, neutrophils, and T lymphocytes (specially Th1 and Th17) are stimulated (34, 35). This response is not severe enough to produce an acute disease picture, as in infection or acute inflammatory response. However, chronic subclinical inflammation may cause relatively higher neutrophil and monocyte levels, as well as platelet levels, which act as acute-phase reactants, compared to the normal population (10, 11, 36–38). There may be functional changes and shifts in the proportions of lymphocyte subgroups (e.g., T cells and B cells); however, a significant increase in the total lymphocyte count is generally not observed, or a relatively smaller increase is seen (39–41). Although there was a relative increase in neutrophil, monocyte, and platelet counts, when evaluated individually, their values remained below the pathological thresholds. Therefore, rather than representing absolute leukocyte elevations, composite indices such as SII, SIRI, and AISI may reflect subtle shifts in immune cell balance accompanying obesity-related metaflammation. Beyond merely reflecting the basic inflammatory burden, composite indices such as the SII, SIRI, and AISI may also indicate the nature of the immune response associated with obesity. While these indices evaluate neutrophils and monocytes associated with adipose tissue macrophage infiltration and metaflammation, along with platelets reflecting endothelial activation, they highlight a more pronounced increase in these cells relative to lymphocytes, which predominantly represent the adaptive immune response. This suggests that the inflammatory response in pediatric obesity is characterized more by innate immune system activation, pointing to a process of immune remodeling in which the adaptive immune response does not show a significant cellular increase.

In our study, we found that multi-component indices, such as SII, SIRI, and AISI, showed stronger associations with obesity severity than traditional indicators, such as NLR and PLR. Adult studies have reported that these new-generation inflammatory indices reflect the degree of inflammation and disease severity in cardiometabolic diseases, diabetes, and metabolic syndrome more sensitively (14, 16–18, 42). Although pediatric data are limited, Luo et al. found that as the body mass index of children and adolescents increased, the levels of SII and SIRI also gradually increased, with the highest values seen in obese children (20). In a study by Li et al. examining the relationship between NLR, PLR, SII, SIRI, and obesity, both SII and SIRI were found to be independently associated with an increased risk of obesity in children and adolescents, and higher values of these indices were consistently linked to a greater likelihood of obesity. While NLR showed a significant increasing trend in obesity and PLR showed a reverse trend, the association for both weakened in regression models and became statistically insignificant (21). In another study focusing on the relationship between AISI and three body composition parameters, including visceral adipose tissue area, higher AISI values were associated with a larger visceral adipose tissue area. Although this study did not examine obesity as a direct outcome, its findings indicate that systemic inflammation is strongly associated with higher visceral fat accumulation and unfavorable body composition in adolescents (23). Our study evaluated a broader range of inflammatory indices across four different BMI categories, including morbid obesity, and demonstrated that these indices are distinctive, especially in the morbid obesity phenotype. Correlation analysis also revealed associations between BMI and the SII, SIRI, and AISI indices. Furthermore, the increasing trend in these indices exhibited a linear rise in the direction of overweight, obese, and morbidly obese. Our findings support the view that pediatric obesity is accompanied by low-grade systemic inflammation and that inflammatory burden appears to increase with obesity severity. However, the relatively low explained variance in the regression models indicates that these hematological indices reflect only one component of the inflammatory process and do not fully represent the multifactorial nature of metabolic inflammation.

In the literature, pediatric studies examining the relationship between inflammatory indices and obesity-related metabolic complications, particularly insulin resistance and metabolic syndrome, have begun to be published in recent years. In their study, Okuyan et al. found that NLR, PLR, and SII were significantly higher in patients with insulin resistance than in those without; however, SIRI and AISI parameters were not evaluated (24). In the study conducted by Gayret et al., the correlation between SII, SIRI, and HOMA-IR was not directly examined; however, it was demonstrated that both SII and HOMA-IR, which are indicators of insulin resistance, were statistically significant risk factors for the presence of metabolic syndrome (25). In the study by Nicoara et al., obese children were divided into those with and without metabolic syndrome, and it was shown that children in the group with metabolic syndrome had significantly higher SII and SIRI values than those in the group without metabolic syndrome (19). Consistent with these findings, SII, SIRI, and AISI were higher in the insulin resistance group in our unadjusted analyses, whereas NLR and PLR were not associated with insulin resistance. However, after adjustment for age, sex, pubertal status, and BMI-SDS, these associations were attenuated. This pattern suggests that the relationship between inflammatory indices and insulin resistance is largely shared with adiposity rather than representing a clearly separable effect. Considering that insulin resistance biologically accompanies adipose tissue dysfunction and metaflammation, models including HOMA-IR were interpreted as describing adjusted associations rather than independence. Therefore, attenuation after adjustment should be understood as reflecting overlapping pathophysiological processes rather than absence of biological relevance.

In our study, in addition to hemogram-based indices used to assess obesity-related inflammatory processes, the MHR index, which also includes HDL in its formulation, was analyzed. It is well established that low levels of HDL cholesterol independently increase the risk of developing insulin resistance, diabetes, and metabolic syndrome (43, 44). In recent years, only a limited number of studies have examined pediatric obesity and its complications using the MHR have been published. Marra et al. found that MHR values were significantly higher in children and adolescents with severe obesity and metabolic syndrome than in those without metabolic syndrome (45). Another recently published study reported that in prepubertal obese children, the MHR was the most sensitive and specific hematological index for detecting clinical features associated with metabolic syndrome (46). Conversely, Türkkan et al. found no difference in the MHR index between 30 obese adolescents and 30 healthy controls, concluding that MHR may not be a reliable biomarker for adolescent obesity (47). In our study, both an increase in BMI-SDS and the presence of insulin resistance, a metabolic complication of obesity, were associated with elevated MHR levels. Unlike other inflammatory indices, this relationship persisted even after adjusting for age, sex, pubertal status, and BMI-SDS. This finding suggests that, unlike hemogram-based indices, the MHR may partially reflect the metabolic changes associated with insulin resistance. However, the limited effect size indicates that the MHR alone is not sufficient for clinical assessment. Future studies could be valuable in investigating whether composite indices that combine lipid parameters with hematological markers contribute to the evaluation of obesity-related metabolic complications.

Clinically, the SII, SIRI, AISI, and MHR indices offer practical advantages owing to their low cost and accessibility from routine laboratory tests. However, ROC analyses demonstrated only modest discriminatory performance (AUC <0.70), indicating that these indices are insufficient as standalone diagnostic tools. Accordingly, they should be interpreted only as complementary biomarkers reflecting inflammatory burden within a broader clinical and metabolic assessment framework.

The main strengths of our study are the use of routine clinical practice data and the examination of a pediatric sample encompassing four different obesity categories: normal weight, overweight, obese, and morbidly obese. Evaluating obesity not only in terms of anthropometric measurements but also together with insulin resistance, a fundamental metabolic complication, has allowed for the pathophysiological interpretation of inflammatory indices. The combined use of trend analysis, correlation analysis, and predefined multivariate regression models based on clinical rationale enabled an assessment of the extent to which the relationships persisted after adjustment for relevant covariates. Furthermore, the clinical discriminative performance of inflammatory indices was evaluated using ROC analyses, contributing to the interpretation of these parameters as complementary biomarkers rather than as standalone diagnostic tools. The joint assessment of multiple hematological inflammatory indices, such as NLR, PLR, SII, SIRI, AISI, and MHR, which are mostly examined separately in the literature, within the same analytical framework, is also a significant methodological strength of this study.

This study has several limitations. The retrospective and cross-sectional design precludes causal inference. The single-center setting may limit generalizability, and the recruitment of the normal-weight control group from a tertiary pediatric endocrinology clinic rather than the general population may have introduced selection bias. The fact that the normal-weight control group was selected from a tertiary pediatric endocrinology outpatient clinic, rather than a community-based sample, may limit external validity. Even if the children presenting to these centers have not received a diagnosis, their healthcare-seeking behavior, frequency of screening, and referral characteristics may differ from the general pediatric population. Therefore, the findings should not be directly generalized to asymptomatic children identified through community screening; instead, they should be interpreted as reflecting the pediatric population that underwent clinical evaluation. In addition, exclusion of conditions such as anemia and vitamin deficiencies, which directly influence hematological indices, may have further affected representativeness. Missing data were handled using a complete-case approach, which may have reduced sample size and introduced additional bias. Because of the retrospective design, an *a priori* sample size calculation was not performed; therefore, findings should be interpreted considering effect sizes and confidence intervals. Information on physical activity and dietary habits was not available and residual confounding cannot be excluded. Moreover, potential non-linear relationships between adiposity and inflammatory indices were not specifically modeled and warrant further investigation. Finally, although ROC analyses were performed, the modest discriminative performance indicates that these indices are not suitable as standalone diagnostic tools. Future multicenter prospective studies are needed to evaluate temporal changes in inflammatory indices and their predictive value for metabolic outcomes.

In our study, inflammatory indices such as SII, SIRI, AISI, and MHR were associated with obesity severity, consistent with their role as markers reflecting the inflammatory burden accompanying pediatric obesity. These findings should therefore be interpreted within a biological continuum linking adiposity, metaflammation, and insulin resistance rather than as indicating independent effects. However, given their limited explanatory power and modest discriminative performance, these indices should not be considered standalone clinical decision tools. Rather, they may be interpreted as supportive biomarkers within a broader clinical and metabolic context. Prospective longitudinal studies are required to clarify their temporal behavior and potential value during follow-up, particularly in relation to changes in adiposity and metabolic status.

## Data Availability

The datasets generated and/or analyzed during the current study are not publicly available due to patient confidentiality and institutional regulations but are available from the corresponding author upon reasonable request and with permission of the relevant ethics committee.
