# Supplementary material for: Evaluation of novel inflammatory biomarkers in overweight, obese, and morbidly obese children: a cross-sectional study
Source: Front Endocrinol (Lausanne). 2026 Mar 11;17:1778022. doi: 10.3389/fendo.2026.1778022 (PMC13012923; doi:10.3389/fendo.2026.1778022)
Supplement: Supplementary file 1 [file Table1.docx]

**Supplementary Table S1. Prevalence of insulin resistance across BMI categories**

| **Insulin resistance** | **Normal weight (n=121)** | **Overweight (n=91)** | **Obese (n=113)** | **Morbid obese (n=92)** | **Total (n=417)** |
| --- | --- | --- | --- | --- | --- |
| **Absent** | **109 (90.1%)** | **47 (51.6%)** | **47 (41.6%)** | **23 (25.0%)** | **226 (54.2%)** |
| **Present** | **12 (9.9%)** | **44 (48.4%)** | **66 (58.4%)** | **69 (75.0%)** | **191 (45.8%)** |

**Supplementary Table S2. Multivariable linear regression analyses of log-transformed inflammatory indices in prepubertal children**

| **Variable** | **ln(SII) β (95% CI)** | **p value** | **ln(AISI) β (95% CI)** | **p value** | **ln(SIRI) β (95% CI)** | **p value** | **ln(MHR) β (95% CI)** | **p value** |
| --- | --- | --- | --- | --- | --- | --- | --- | --- |
| BMI-SDS | −0.001 (−0.058–0.057) | 0.995 | 0.052 (−0.070–0.117) | 0.621 | 0.013 (−0.084–0.095) | 0.903 | 0.148 (−0.035–0.152) | 0.215 |
| HOMA-IR | 0.440 (0.049–0.140) | <0.001 | 0.356 (0.046–0.195) | 0.002 | 0.224 (−0.002–0.140) | 0.055 | 0.102 (−0.031–0.075) | 0.413 |
| Age | 0.024 (−0.040–0.051) | 0.810 | 0.028 (−0.064–0.083) | 0.790 | 0.067 (−0.048–0.092) | 0.537 | 0.211 (−0.009–0.108) | 0.098 |
| Sex | −0.058 (−0.206–0.109) | 0.544 | −0.012 (−0.272–0.242) | 0.907 | −0.053 (−0.308–0.181) | 0.609 | −0.193 (−0.354–0.036) | 0.108 |

*All dependent variables were log-transformed prior to analysis due to skewed distributions. Multivariable linear regression models were pre-specified based on clinical rationale and constructed using the enter method. All models were adjusted for BMI-SDS, HOMA-IR, age, and sex. Variance inflation factors were <2.5 for all models, indicating no relevant multicollinearity.*

**Supplementary Table S3. Multivariable linear regression analyses of log-transformed inflammatory indices in pubertal children**

| **Variable** | **ln(SII) β (95% CI)** | **p value** | **ln(AISI) β (95% CI)** | **p value** | **ln(SIRI) β (95% CI)** | **p value** | **ln(MHR) β (95% CI)** | **p value** |
| --- | --- | --- | --- | --- | --- | --- | --- | --- |
| BMI-SDS | 0.236 (0.032–0.129) | 0.001 | 0.127 (−0.010–0.140) | 0.090 | 0.074 (−0.034–0.103) | 0.323 | 0.154 (0.002–0.116) | 0.044 |
| HOMA-IR | −0.063 (−0.020–0.008) | 0.386 | 0.031 (−0.017–0.026) | 0.674 | 0.046 (−0.013–0.026) | 0.537 | 0.177 (0.002–0.028) | 0.021 |
| Age | −0.023 (−0.030–0.021) | 0.732 | −0.035 (−0.050–0.030) | 0.620 | 0.039 (−0.026–0.046) | 0.581 | −0.080 (−0.041–0.012) | 0.274 |
| Sex | −0.157 (−0.297–−0.023) | 0.022 | −0.067 (−0.316–0.110) | 0.340 | −0.035 (−0.244–0.145) | 0.616 | 0.116 (−0.028–0.250) | 0.117 |

*All dependent variables were log-transformed prior to analysis due to skewed distributions. Multivariable linear regression models were pre-specified based on clinical rationale and constructed using the enter method. All models were adjusted for BMI-SDS, HOMA-IR, age, and sex. Variance inflation factors were <2.5 for all models, indicating no relevant multicollinearity.*

**Supplementary Table S4. Multivariable linear regression analyses of log-transformed inflammatory indices adjusted for BMI-SDS, age, sex and pubertal status (without HOMA-IR)**

| **Variable** | **ln(SII) β (95% CI)** | **p value** | **ln(AISI) β (95% CI)** | **p value** | **ln(SIRI) β (95% CI)** | **p value** | **ln(MHR) β (95% CI)** | **p value** |
| --- | --- | --- | --- | --- | --- | --- | --- | --- |
| **BMI-SDS** | 0.067 (0.035–0.099) | **<0.001** | 0.083 (0.034–0.132) | **0.001** | 0.062 (0.017–0.107) | **0.007** | 0.074 (0.031–0.117) | **0.001** |
| **Age (years)** | 0.016 (−0.005–0.037) | 0.132 | 0.023 (−0.009–0.054) | 0.160 | 0.031 (0.002–0.060) | 0.039 | −0.001 (−0.025–0.023) | 0.933 |
| **Sex** | −0.072 (−0.177–0.032) | 0.174 | −0.010 (−0.168–0.149) | 0.904 | −0.014 (−0.160–0.132) | 0.853 | 0.059 (−0.051–0.170) | 0.292 |
| **Pubertal status** | 0.041 (−0.109–0.191) | 0.591 | 0.063 (−0.165–0.290) | 0.589 | 0.055 (−0.155–0.265) | 0.607 | 0.077 (−0.089–0.242) | 0.362 |

*All dependent variables were log-transformed prior to analysis due to skewed distributions. Multivariable linear regression models were pre-specified based on clinical rationale and constructed using the enter method. Variance inflation factors were <2.5 for all models, indicating no relevant multicollinearity.*
