# Supplementary figures and images for: Evaluation of novel inflammatory biomarkers in overweight, obese, and morbidly obese children: a cross-sectional study
Source: Front Endocrinol (Lausanne). 2026 Mar 11;17:1778022. doi: 10.3389/fendo.2026.1778022 (PMC13012923; doi:10.3389/fendo.2026.1778022)

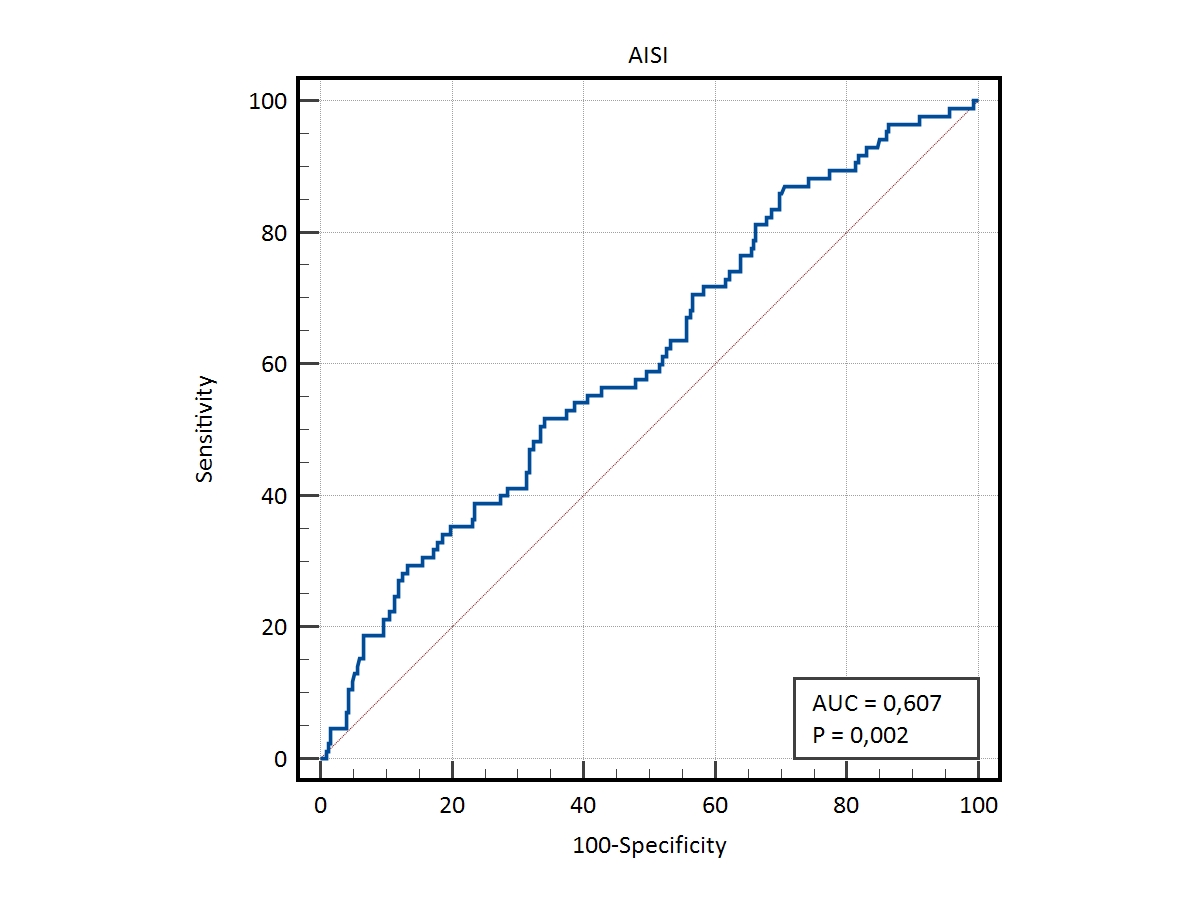

Supplement: Supplementary file 2 [file Image1.jpeg]

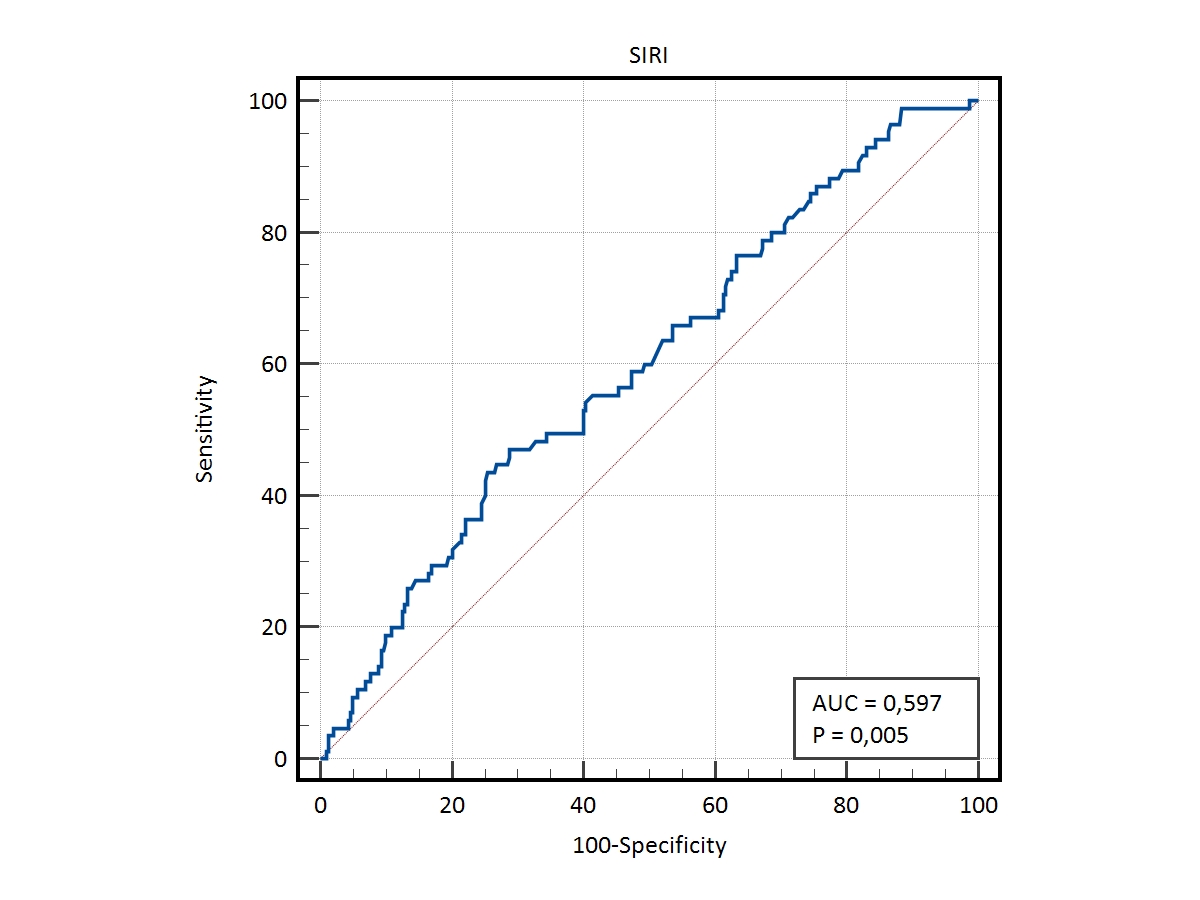

Supplement: Supplementary file 3 [file Image2.jpeg]

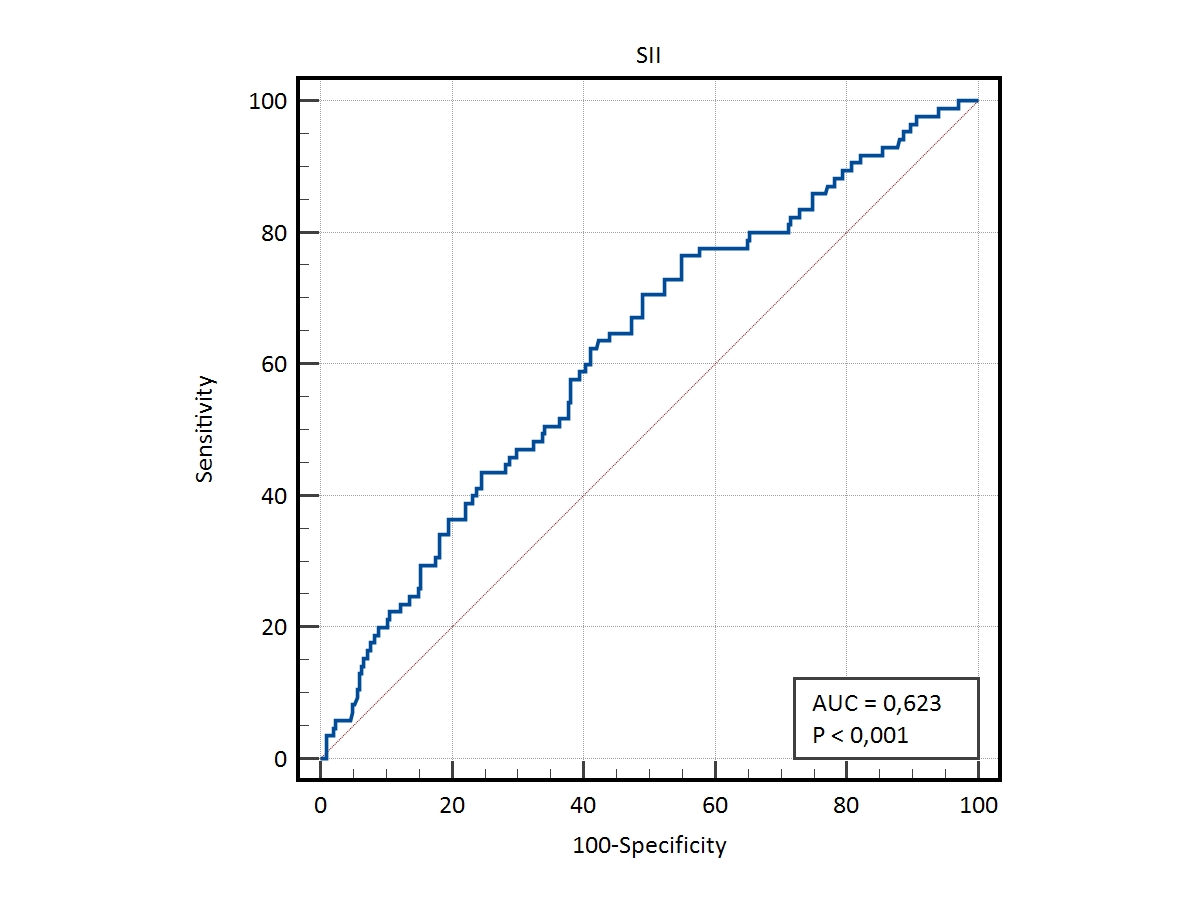

Supplement: Supplementary file 4 [file Image3.jpeg]

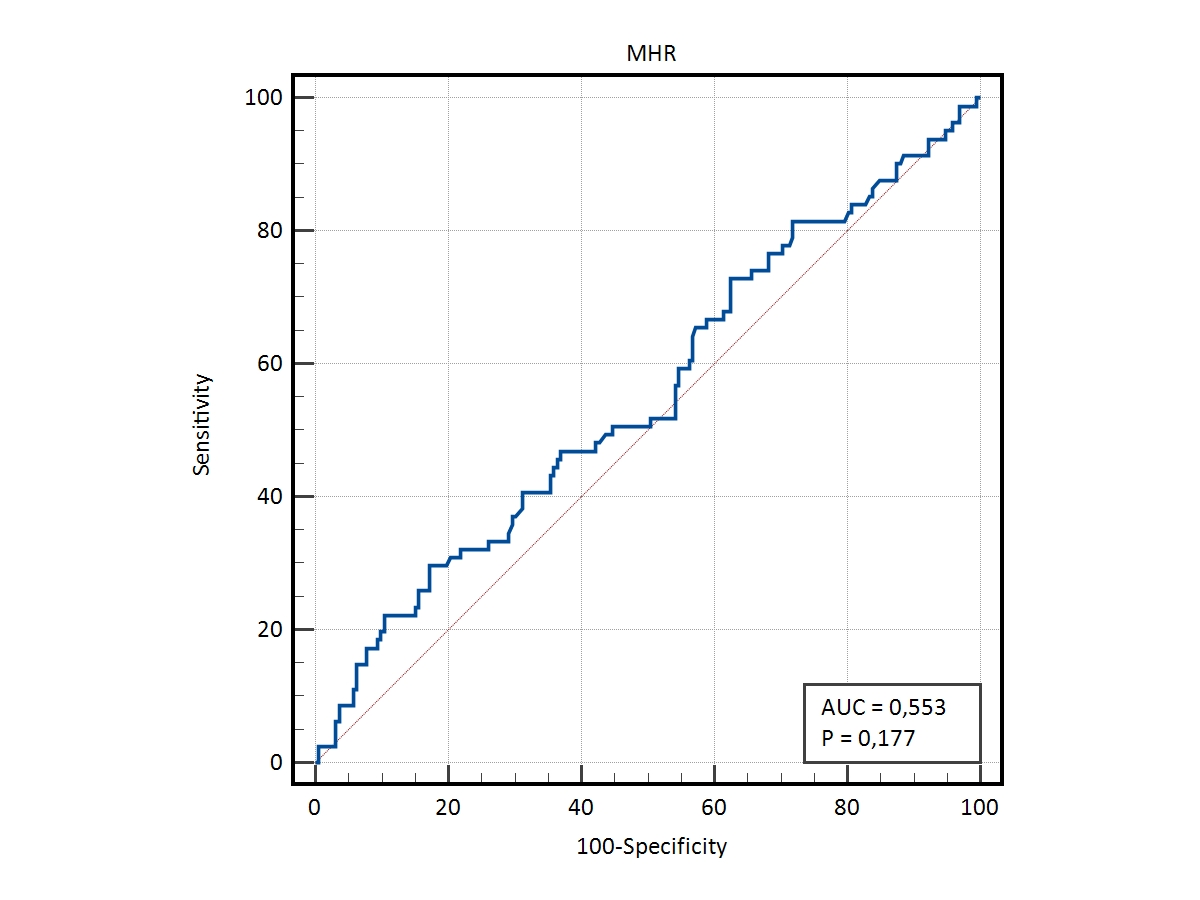

Supplement: Supplementary file 5 [file Image4.jpeg]

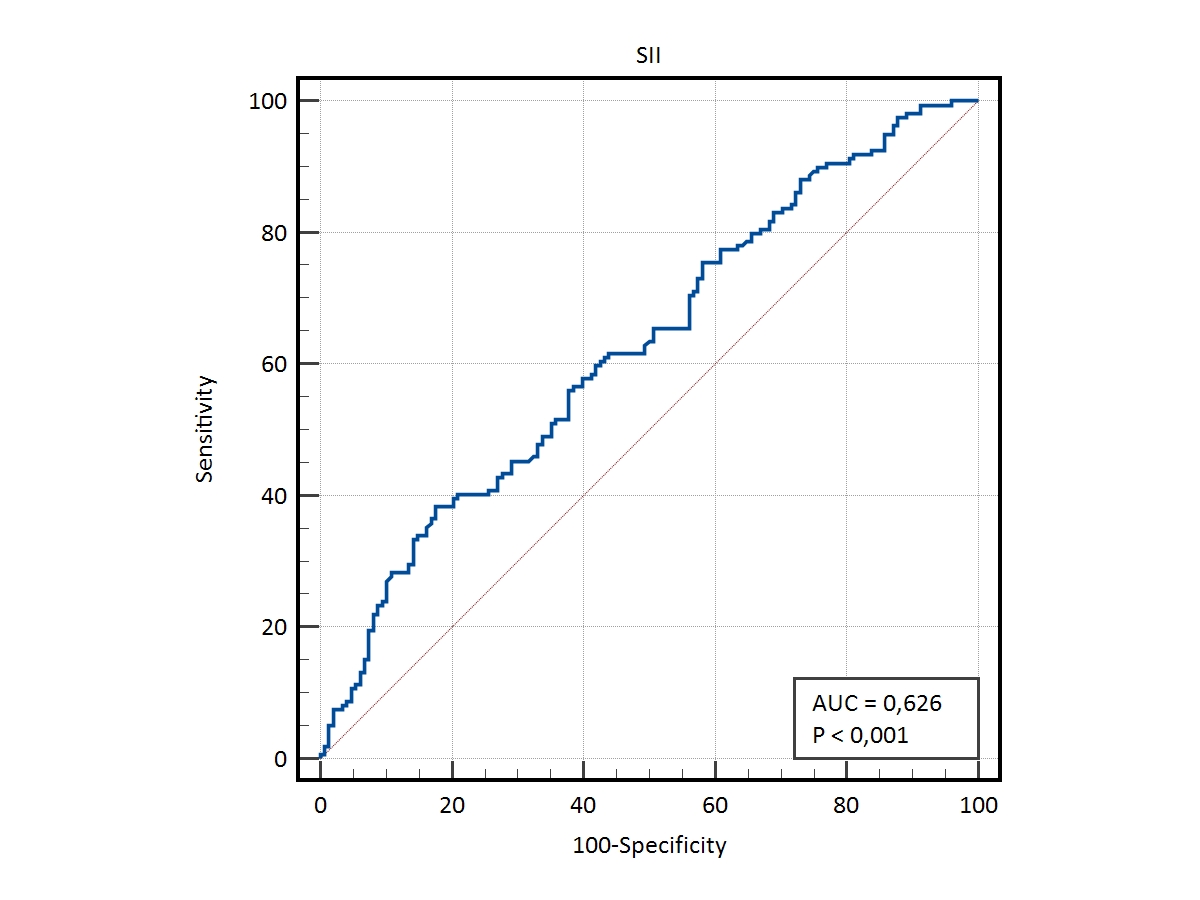

Supplement: Supplementary file 6 [file Image5.jpeg]

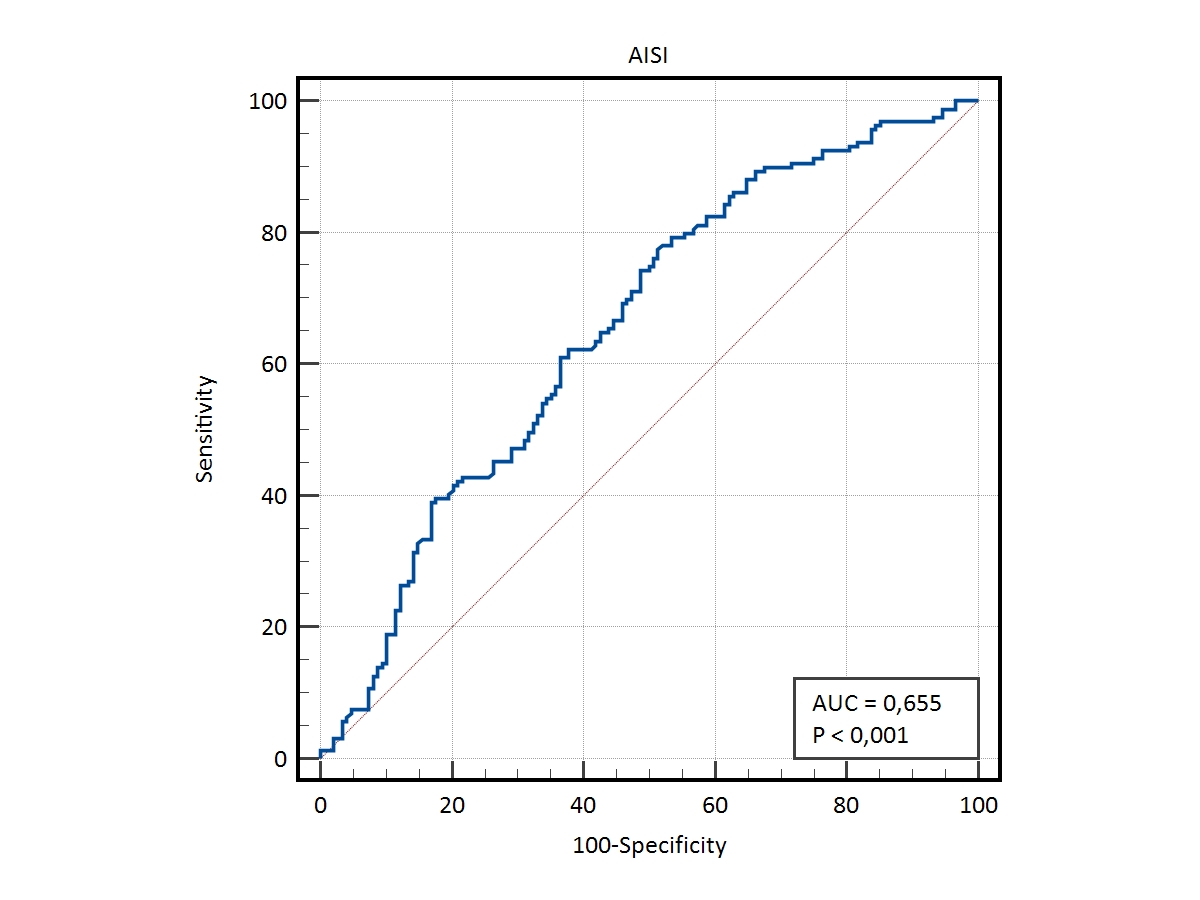

Supplement: Supplementary file 7 [file Image6.jpeg]

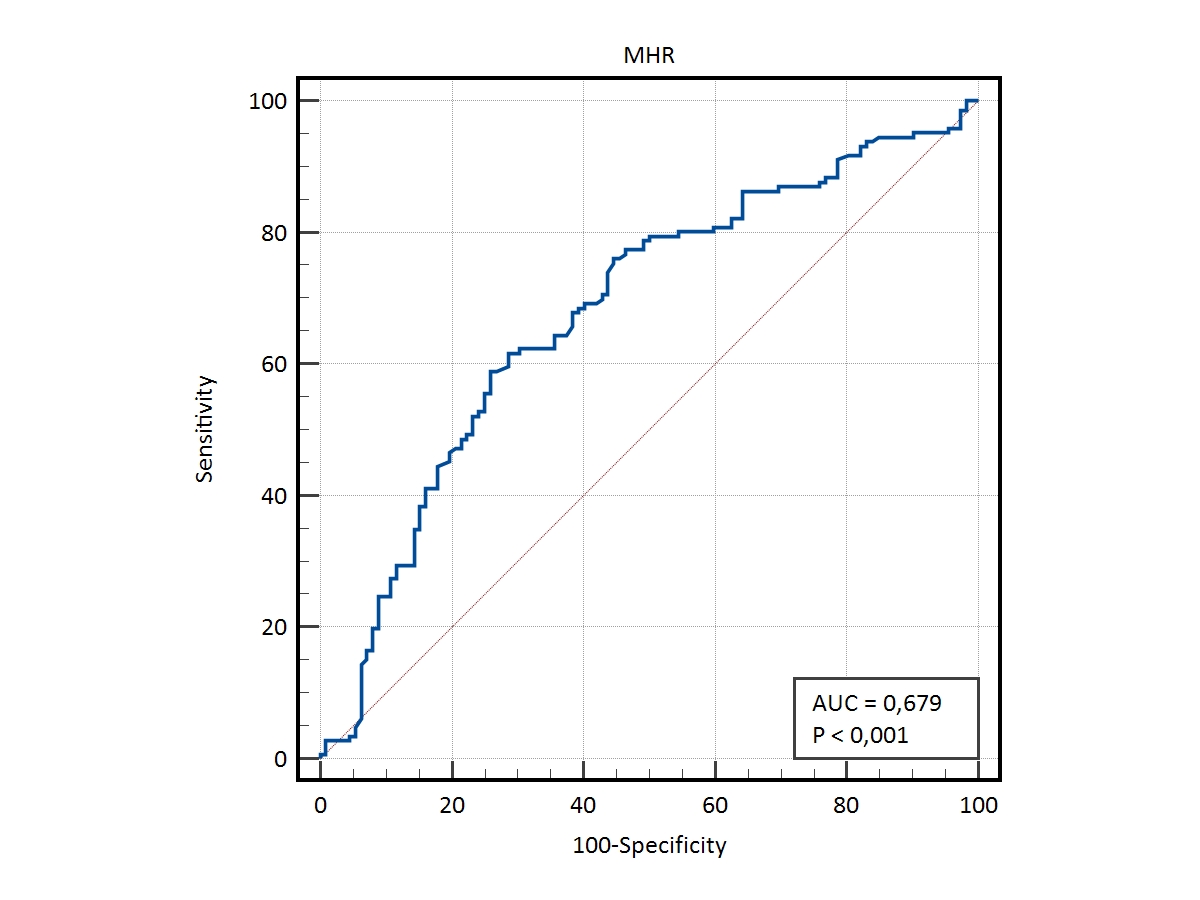

Supplement: Supplementary file 8 [file Image7.jpeg]

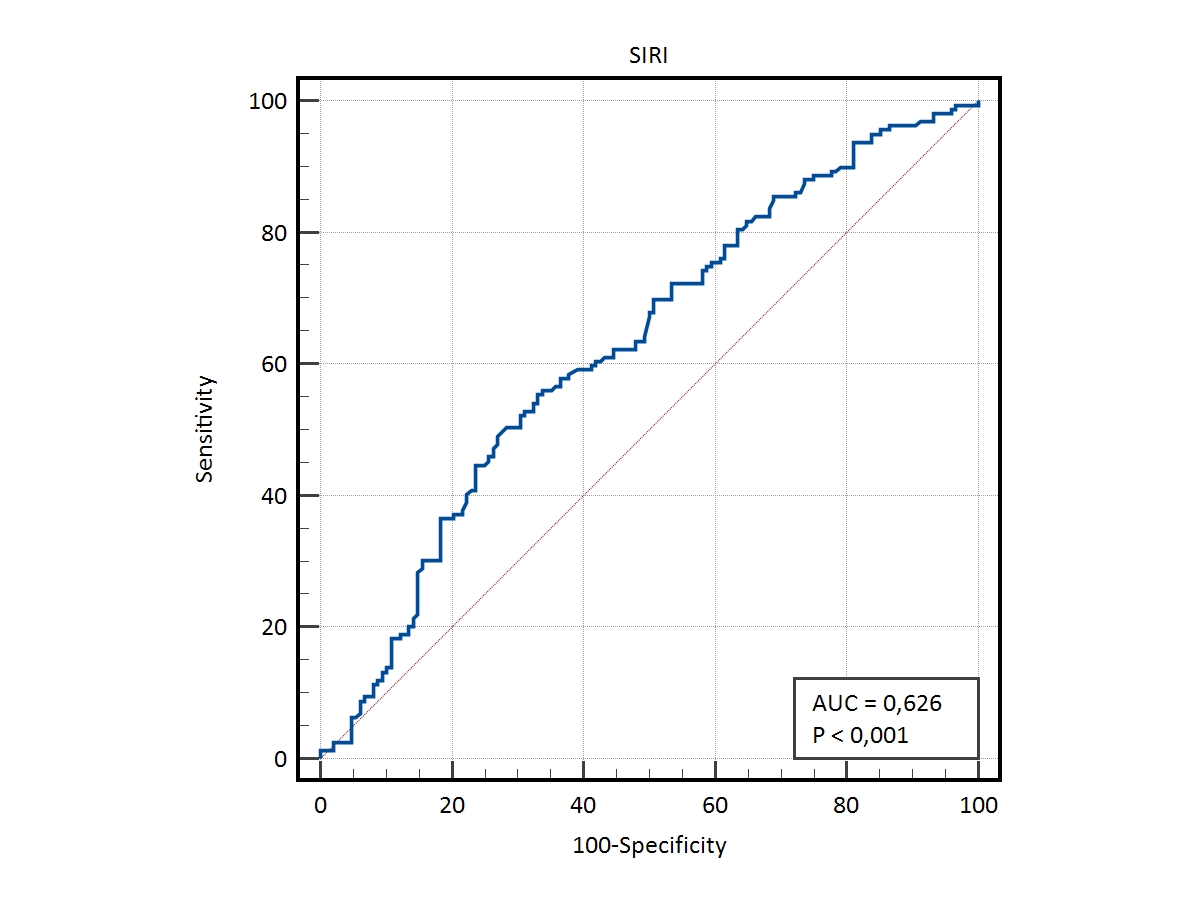

Supplement: Supplementary file 9 [file Image8.jpeg]
